# Young Adult E-Cigarette and Combustible Tobacco Users Attitudes, Substance Use Behaviors, Mental Health, and Neurocognitive Performance

**DOI:** 10.3390/brainsci12070889

**Published:** 2022-07-06

**Authors:** Natasha E. Wade, Kelly E. Courtney, Neal Doran, Rachel Baca, Laika D. Aguinaldo, Courtney Thompson, Jamie Finegan, Joanna Jacobus

**Affiliations:** Department of Psychiatry, University of California, San Diego, CA 92037, USA; nwade@health.ucsd.edu (N.E.W.); kecourtney@health.ucsd.edu (K.E.C.); nmdoran@health.ucsd.edu (N.D.); rebaca@health.ucsd.edu (R.B.); laaguinaldo@health.ucsd.edu (L.D.A.); crthompson@health.ucsd.edu (C.T.); jlfinegan@health.ucsd.edu (J.F.)

**Keywords:** young adults, neurocognition, e-cigarettes, cigarettes, nicotine attitudes, depression

## Abstract

Nicotine and tobacco product (NTP) use has escalated, largely due to the advent of e-cigarettes. The NTP administration method (i.e., combustible cigarette, e-cigarette) may be an important differentiator. We assessed young adult substance use history, nicotine attitudes, mental health, and neurocognition by the NTP use method. Emerging adults (16–22 year olds) were divided into combustible NTP users (Combustible+ = 79, had used any combustible NTP in the last 6 months), non-combustible users (E-Cig = 43, had used non-combustible NTP, in the past 6 months), and NTP Naïve (*n* = 79; had not used NTP in the past 6 months) based on past 6-month NTP use patterns. Participants completed self-report and objective neurocognition measures. Analysis of covariance assessed mental health and neurocognition by group, controlling for confounds and correcting for multiple comparisons. Nicotine groups reported more favorable attitudes toward combustible cigarette and e-cigarette use, with taste as the primary reason for e-cigarette use. Combustible+ reported more nicotine dependence and craving. Substance use differed by group, with Combustible+ using the most NTP, alcohol, and cannabis. Nicotine groups reported higher depression and stress symptoms; male Combustible+ reported higher depression symptoms than other same-gender groups. Groups did not differ on neurocognition, though cannabis use was associated with inaccurate emotional Stroop responses. Overall, research suggests that young adult combustible users are likely qualitatively different from non-combustible users. Understanding the unique characteristics related to NTP product use will help guide intervention and prevention development.

## 1. Introduction

Adolescent and young adult nicotine use has experienced a resurgence in the past decade with the rise in popularity of electronic nicotine delivery systems (ENDS, commonly referred to as vaping or e-cigarettes), with 4% of 12th graders smoking cigarettes in the past month and 20% vaping nicotine in 2021 (versus a peak of 37% of 12th graders smoking cigarettes in the past month in 1997 [1]). Once initiated, the use of nicotine and tobacco products (NTP) may become a persistent issue for emerging adults, as e-cigarette use increases the odds of combustible cigarette consumption [2,3]. Despite a plateau in overall substance use prevalence during the COVID-19 pandemic (e.g., 35% of 12th graders vaped nicotine in the past year in 2020 versus 27% in 2021), and evidence of fewer individuals initiating use during the pandemic as compared to recent pre-pandemic years [1], the 2021 National Youth Tobacco Survey still found that NTP use is a serious public health issue for youth. During the global pandemic, more than 2 million middle schoolers and high schoolers identified as current e-cigarette users and 44% of high schoolers using e-cigarettes reported use on >20 out of 30 days [4]. Further, the vast majority of emerging adults ages 16–24 reported maintaining or increasing their e-cigarette or cigarette use during the pandemic [5]. With this rapidly evolving landscape of combustible and non-combustible NTP use, greater understanding is needed of the unique relationships between motivations for NTP use, modes of use, and behavioral outcomes that can contribute to the development of timely and relevant prevention and intervention approaches.

Initiation of NTP use in adolescence and young adulthood is particularly concerning, given the vast neurodevelopmental changes occurring in this life stage. Changes in gray and white matter tissue subserve neural maturation and specialization and contribute to complex thinking abilities and efficient neurocognitive processing [6,7]. Neuromaturational changes are regionally specific and vary in timing across adolescence and young adulthood [8,9]; they may leave some youth susceptible to the rewarding effects of substances such as nicotine [10,11], and to downstream cognitive and mental health deficits [3,12,13]. The dynamic changes occurring in brain circuitry underlying motivation, reward, and control underscore the importance of investigating how NTP use, and what levels of NTP use, may influence brain structure during unique developmental periods.

There have been conflicting findings regarding the neurodevelopmental correlates of NTP use in youth. In one of our first papers focused on the co-use of nicotine and cannabis [14], our team found that higher levels of urinary cotinine (nicotine’s major metabolite, mean concentration = 89.3 ng/mL) significantly predicted decreased memory performance among both single substance users and co-users of cannabis and nicotine ages 16–22. In a follow-up paper examining differences in structural brain metrics with an overlapping yet larger sample of the same age, we found no evidence of poorer white matter tissue and blood flow integrity among co-users of nicotine and cannabis (*n* = 53–55), although there was evidence of significantly poorer brain health outcomes among cannabis only users [15,16]. Others also failed to find poor structural brain integrity in young adult nicotine smokers [17,18]. One study found significantly decreased hippocampal volume in cannabis and NTP co-users, but that decreased volume related to significantly better memory performance [19]. Notably, frequency and amount of cannabis and nicotine use varied by study. Thus, it remains unclear whether nicotine counteracts some of the effects of cannabis-related neural alterations early in life, or if observations are revealing unique brain phenotypes of co-users, or both.

It may be that some of these disparate findings are due to different modes of administration of NTP (e.g., e-cigarette use versus combustible products) that is not typically well assessed, as research comparing combustible versus non-combustible NTPs is limited. Indeed, only two studies have considered the specific impact of e-cigarette use on cognition, finding that both adolescents and adults self-report cognitive difficulties [20,21]; however, no objective measurement of cognition has been reported to date. A growing body of literature also suggests that the use of combustible cigarettes may be particularly important, as it is linked to significantly more severe nicotine dependence and other behavioral outcomes [22,23,24], which may in turn impact neurodevelopment. Finally, there is also evidence of mental health [25] and attitudinal [26] differences between e-cigarette and combustible NTP users, though this has not been studied extensively or in concert with overall attitudes, substance use history, neurocognition, and mental health.

Here, we assessed NTP use attitudes, mental health, and neurocognition in NTP users ages 16–22. We focus on exploring differences in those who used combustible products within the past six months, compared to e-cigarette only users and individuals who did not use any NTP. We first describe and examine differences in substance use history, motivations for nicotine use, and attitudes by nicotine product user group. We then investigate differences in mental health and neurocognitive performance by nicotine group status (i.e., combustible versus e-cigarette only users). In each instance, we expected both nicotine groups to exhibit more cumulative substance use [24], stronger positive attitudes toward nicotine use, more mental health distress, and poorer neurocognitive performance than NTP naïve participants [20,21]. Given that we anticipated increased dependence and substance use among our combustible users, we hypothesized that within each of these domains, combustible product users would demonstrate poorer outcomes as compared to e-cigarette only users [22,24].

## 2. Materials and Methods

Participants: Participants (*n* = 203) included young adults aged 16 to 22 in an ongoing study of the effects of cannabis and nicotine use on adolescent brain development. Participants were recruited via flyers posted at high schools, universities, and community colleges, as well as postings on social media sites. Interested individuals contacted the laboratory through an email or phone call to schedule an initial screening interview to determine eligibility. Individuals aged 18 years or older provided verbal informed consent prior to the screening. For those younger than 18, permission was obtained from their parent or legal guardian before receiving verbal assent to participate from the youth. The screening interview consisted of questions related to their substance use, medical, and mental health histories.

Sociodemographics: Participants self-reported age, gender, education, race, and ethnicity. Mother’s education level was used as a proxy for socioeconomic status. We note that sociodemographic characteristics often are social constructs and proxies for other factors which need to be appropriately contextualized [27,28]. Therefore, while sociodemographics are important to describe a sample, given the relatively small sample and restricted range of sociodemographic characteristics presented here, comprehensive analysis of appropriate contextualizing factors is not possible. We therefore will present the data but limit interpretation.

Inclusion criteria: Group status was based on 6-month history of combustible product or NTP use and defined as follows. NTP-Naïve participants had used no NTP products in their lifetime (*n* = 79); E-Cig-only users reported they used e-cigarettes and had not used combustible NTP products in the past six months (*n* = 45); Combustible+ had used combustible NTPs within the past six months (*n* = 79). The E-Cig group was so named, as all participants reported e-cigarette use in the past six months. Of note, 82% of Combustible+ had also used e-cigarettes in the past month. Non-combustible NTP use was defined as use of any of the following products: electronic cigarettes (e.g., vape pens, and Juul), smokeless tobacco (e.g., chew, snuff, and snus), and nicotine replacement products (e.g., patches, gum, nasal sprays, inhalers, and lozenges). Combustible NTP use included tobacco cigarettes, tobacco pipe, hookah with tobacco, and cigars; cigarettes were the primary type of combustible product used, with 73% of Combustible+ reporting cigarette use in the past six months. Cannabis and alcohol users were included across groups, and levels of cannabis and alcohol use covaried as relevant in subsequent analyses.

Exclusion criteria: Exclusion criteria included a history of major medical or neurological issues; past major head injuries; past or current DSM-5 diagnoses other than cannabis use disorder, nicotine use disorder, or anxiety and depression disorders (with no previous or current medication use); learning and developmental disorders; use of medications that may affect the brain; excessive alcohol use (>2 drinking occasions/week); excessive prenatal exposure to alcohol (maternal use of >2 drinks per occasion or >4 drinks in a week), drugs, or tobacco; other gestational or birth complications, including premature birth (≥6 weeks early) and low birth weight (<5 lbs); hearing or vision problems that were non-correctable; MRI contraindications, including implanted/irremovable metallic objects and pregnancy; and intoxication (alcohol or cannabis) at the time of their study session (assessed with breathalyzer and oral fluid toxicology). Participants reported all other substance use, including spice, opiates, amphetamines (other than as prescribed), barbiturates, hallucinogens, cocaine, inhalants, benzodiazepines, MDMA, ketamine, GHB, and PCP.

Procedures. Participation entailed the completion of a four-hour study session, consisting of questionnaires related to substance use and mental health, neurocognitive testing, and a magnetic resonance imaging (MRI) scan (not included in the current analysis). All participants gave written informed consent (≥18 years old) or parental consent and participant assent (<18 years old) before beginning the study session. Prior to their appointment, participants were asked to abstain from all drug use, aside from nicotine, for 12 h. Participants were able to use NTP during the study session to avoid withdrawal effects. Most recent NTP use was recorded during the study session (range = 0–3650 days, median = 4 days). All procedures were approved by the University of California, San Diego Institutional Research Board.

### 2.1. Measures

Substance use history: A modified version of the Customary Drinking and Drug Use Record (CDDR; Brown et al., 1998; Jacobus et al., 2018; Karoly, Schacht, Jacobus, et al., 2019; Karoly, Schacht, Meredith, et al., 2019) was administered to assess quantity and frequency of use of NTP, cannabis, and alcohol. Past six months and lifetime use were measured in terms of independent episodes, allowing for multiple uses to be reported within a single day (e.g., first thing in the morning, again before bed). Participants were asked to provide additional details related to each substance reported, including age at first use, onset of regular (weekly) use, product type, potency (cannabis; scored as 0 = never to sometimes using high potency cannabis, or 1 = most of the time to always), and perceived intensity during use. High potency was defined as >15% THC for flower and >80% for concentrates. Further, urinary analysis was used to assess objective and quantified levels of nicotine and cannabis exposure (see [14] for details).

Mental health: Participants completed self-report measures designed to examine mental health and emotional states. The Beck Depression Inventory [29] (BDI-II) measures the severity level for various physical and psychological symptoms related to depression (e.g., loss of energy, changes in sleeping pattern). A cut-off of 14 was used to indicate a clinical threshold for depression on the BDI. The Depression Anxiety Stress Scale [30] (DASS-21) short form consists of 21 items examining the frequency of negative emotional experiences within the last seven days, with scores on depression, anxiety, and stress subscales. The State-Trait Anxiety Inventory [31] (STAI) was used to measure participants’ state of anxiety on the day of the assessment. Raw scores were used on each scale.

Substance use attitudes: Several additional self-report questionnaires were given to assess motivations for use and outcome expectancies for both combustible and non-combustible NTP. Participants were instructed to provide their opinion regardless of whether they had ever used combustible or non-combustible NTP. They completed an adapted Smoking Consequences Questionnaire [32] (SCQ), with questions specific to e-cigarette use and four subscales calculated: negative consequences, positive reinforcement, negative reinforcement, and weight control. Similarly, both NTP naïve and users completed the Tobacco Motives Inventory [33] (TMI), a 15-item instrument describing different reasons for smoking combustible cigarettes. The 12-item Electronic Cigarette Attitudes Survey [34] (ECAS) was administered to further examine attitudes toward the use of e-cigarettes versus combustible NTP. The Questionnaire of Smoking Urges [35,36] (QSU) and the Hooked on Nicotine Checklist [37] (HONC) were also given to NTP users to acquire information on NTP addiction severity, with total scores calculated.

Neurocognition, NIH Toolbox: Participants completed the National Institutes of Health (NIH) toolbox cognition battery [38], consisting of seven individual tasks. All tests were completed using the NIH Toolbox app on 3rd generation iPad Air devices (10.5 in). Research subjects were seated upright and used their dominant index finger to make each response. To prevent participants from inadvertently skipping through instructions, a one-second touch-and-hold button was required to advance to the next task. Tasks completed included the Picture Vocabulary Task, where participants identified the picture matching the meaning of a word they were read aloud; oral reading, where participants received a single word as visual stimuli written on screen and were asked to read each aloud; the Dimensional Change Card Sort (DCCS) Test, where participants had to respond to stimuli based on changing rules displayed on top of the screen; Flanker Inhibitory Control and Attention Test, where participants had to select a left or right arrow based on a displayed target stimulus arrow in the midst of a row of arrows; the List Sort Working Memory Test was administered to assess working memory and required participants to sort stimuli, presented both visually and auditorily, from smallest to largest in size; Picture Sequence Memory Test, a measure of episodic memory, where participants had to recall a sequence of displayed pictures; and Pattern Comparison Processing Speed Test, where participants had to quickly decide if two side-by-side images were the same or different. Population-adjusted scores, which adjusted for age, gender, race, ethnicity, and education level, were used in the present analyses. For the Rey Auditory Verbal Learning Test [39] (RAVLT), participants were read 15 words over five trials and asked to recall the list after each repetition. A new, second list was read, and then participants were asked to again recall the original list. After 30 min, they were again asked to recall the original list. Raw scores on initial (trial 1) recall, total score over all trials, and long-delay recall were collected. The Game of Dice Task (GDT) [40] assesses decision-making and risk-taking behaviors. Participants view a virtual single die shaking in a cup, guessing one single number (worth USD 1000) or a combination of two, three, or four numbers (worth USD 500, 200, and 100, respectively). A correct prediction would add the specified monetary amount to their total earnings, while an incorrect guess would result in a loss of the same amount. Performance was measured by a net score, computed by subtracting the number of disadvantageous, high-risk choices from the number of advantageous, low-risk decisions. Participants also completed a variation of the Emotional Stroop Task (Emotional Word–Emotional Face Stroop, or EWEFS) [41] as a measure of emotional processing and cognitive control. The presentation of an emotional word (e.g., angry) was overlaid on a picture of a face that is displaying an emotion either congruent or incongruent to the word presented. The stimuli appeared one at a time on screen, and participants worked to sort the words into two categories (bad vs. good) while ignoring the distractor image in the background. Scoring was based on the total correct responses for congruent and incongruent trials.

### 2.2. Analysis Plan

Selection of Covariates: Sociodemographic characteristics (age, gender, race, ethnicity, education, mother’s education as a proxy for socioeconomic status) were considered for inclusion in all analyses. Inclusion of covariates was determined based on characteristics that differed by group. Models with measures that were corrected for sociodemographics (i.e., NIH Toolbox) did not include sociodemographic variables as covariates. For primary outcome analyses of mental health and neurocognition, ANCOVAs were run, controlling for the past six months of alcohol and cannabis use and sociodemographic factors, which differed by group (i.e., age, education, and gender).

Primary analyses: SPSS 28.0 was used for all primary analyses, and [42] and Rstudio [43] were used for multiple comparison corrections using the “sjstats” package for false-discovery rate corrections [44,45]. Differences by group (ANOVAs, chi-squares) in sociodemographics, substance use attitudes, substance use history are first presented. The selection of covariates was determined by ANOVAs and chi-squares. ANCOVAs assessed differences in mental health and neurocognition by group, controlling for alcohol and cannabis use in the past 6 months, and relevant sociodemographic covariates (see above). Benjamini and Hochberg’s false discovery rate [44] method was used within models to correct for multiple comparisons in ANCOVA models. In models with significant differences by NTP group (whether run as ANOVAs or ANCOVAs), Fisher’s post-hoc tests were run to identify specific group differences, and marginal means were reviewed to determine directionality. Given the age range of participants, post-hoc analyses were conducted with only participants 18–22 years old. All models were re-analyzed, with covariates again selected by sociodemographic differences between groups and including past six-month cannabis and alcohol use.

## 3. Results

### 3.1. Participant Characteristics

***Sociodemographics*:** Groups differed by age (F(2,198) = 7.98, *p* < 0.001), education (F(2,198) = 3.08, *p* = 0.048), and gender (χ^2^ = 14.05, *p* < 0.001), but not by race (*p* = 0.06), ethnicity (*p* = 0.52) or mother’s education (*p* = 0.80). See Table 1.

**Figure 1:**
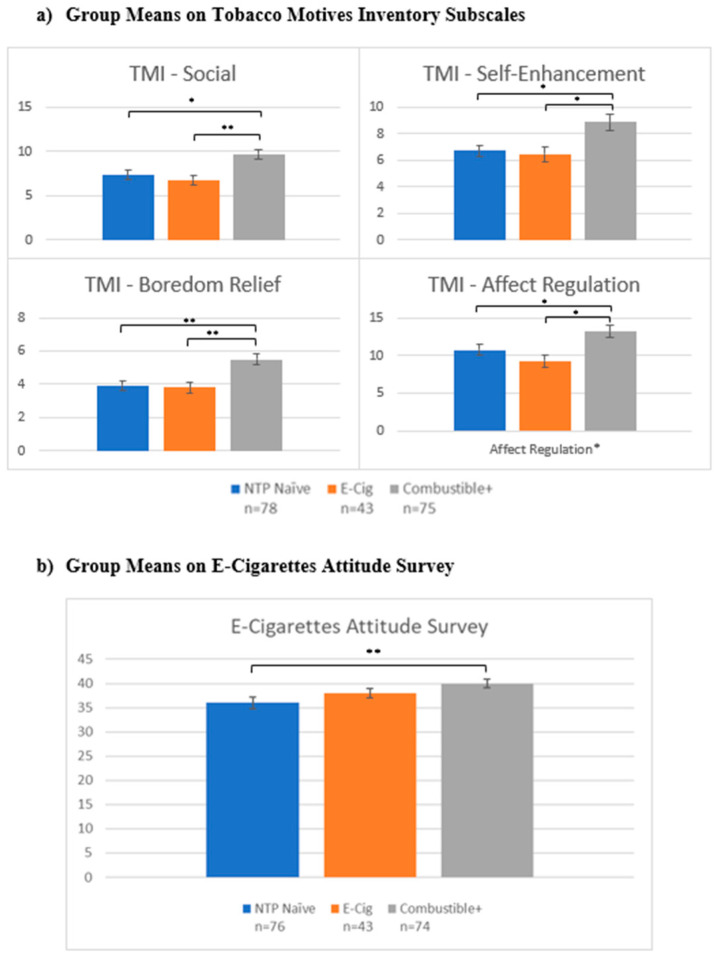
NTP use attitudes and motives. Notes: * denotes *p* < 0.05; ** denotes *p* < 0.001. TMI = Tobacco Motives Inventory; NTP = Nicotine and Tobacco Product. *Y*-axis represent scores on each scale and subscale.

***Motivations, attitudes, and addiction severity*:** Groups differed in response to the Tobacco Motivations Inventory across subscales (social: F(2193) = 8.23, *p* < 0.001; self-enhancement: F(2193) = 6.57, *p* = 0.002; boredom: F(2193) = 9.00, *p* < 0.001; affect regulation: F(2193) = 6.15, *p* = 0.003). Review of marginal means and post-hoc analyses reveal that Combustible+ scored higher than E-Cig (social: *p* < 0.001; self-enhancement: *p* = 0.003; boredom relief: *p* < 0.001; affect regulation: *p* = 0.005) and NTP Naïve (social: *p* = 0.001; self-enhancement: *p* = 0.002; boredom relief: *p* < 0.001; affect regulation: *p* = 0.02), while NTP Naïve did not differ from E-cig on any subscales (see Figure 1a). On the E-Cigarettes Attitude Survey score, group differences were also present (F(2190) = 3.64, *p* = 0.03; Figure 1b), with Combustible+ (*p* = 0.008) reporting higher scores than NTP Naïve, indicating a more favorable attitude toward e-cigarettes, and no difference between E-Cig and NTP Naïve or Combustible+ and E-Cig.

**Figure 2:**
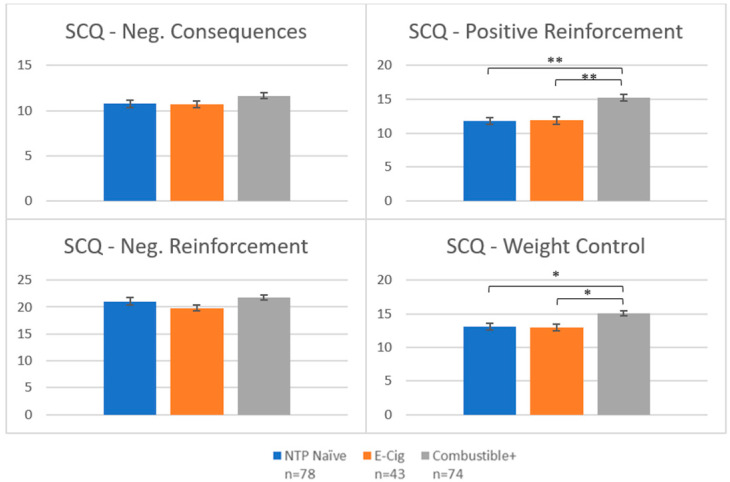
Group means on E-Cigarette Smoking Consequences Questionnaire. Notes: * denotes *p* < 0.05; ** denotes *p* < 0.001. NTP = Nicotine and Tobacco Product. *Y*-axis represents scores on each subscale.

Groups also differed on two e-cigarette-adapted Smoking Consequences Questionnaire (SCQ) subscales: positive reinforcement (F(2192) = 16.53, *p* < 0.001) and weight control (F(2192) = 6.39, *p* = 0.002; see Figure 2). In both cases, Combustibles+ scored higher than NTP Naïve (Positive Reinforcement: *p* < 0.001; Weight Control: *p* = 0.002) and E-Cig (Positive Reinforcement: *p* < 0.001; Weight Control: *p* = 0.005), indicating Combustible+ reported stronger expectancies for sensory satisfaction and weight control. There were no between group differences on SCQ Negative Consequences (*p* = 0.14) or Negative Reinforcement subscales (*p* = 0.08).

**Figure 3:**
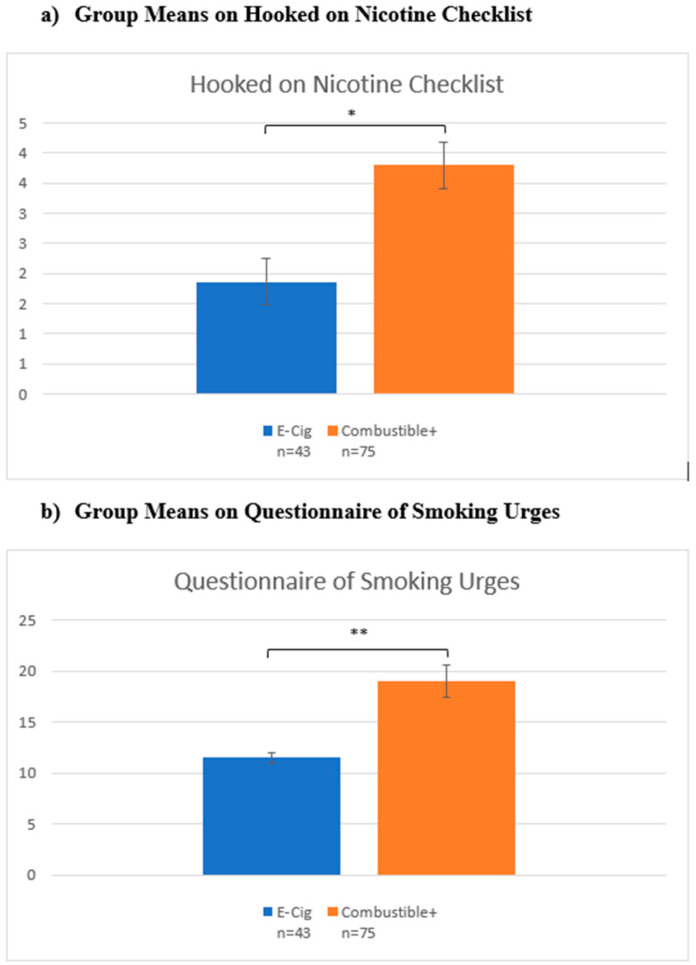
Nicotine dependence and craving questionnaires. Notes: * denotes *p* < 0.05; ** denotes *p* < 0.001. *Y*-axis represents scores on each scale.

Finally, on measures of dependence and craving, Combustible+ scored higher than E-Cig on both the Hooked on Nicotine Checklist (F(1116) = 10.87, *p* = 0.001; Figure 3a), and the Questionnaire of Smoking Urges total score (F(1116) = 12.23, *p* < 0.001; Figure 3b).

***Reasons for E-Cigarette Use*.** Within both nicotine user groups, the most common reason for e-cigarette use was “I prefer the taste of an e-cigarette” (58% of E-Cig, 56% of Combustibles+). Full prevalence for potential reasons of use and differences in prevalence between group by chi-square are presented in Table 2.

***Urinary detected substance use:*** No NTP-Naïve individuals had cotinine, the primary metabolite of nicotine, present in their urine. A total of 40% of E-Cig and 38% of Combustible+ had cotinine present in their urine. In addition, 30% of NTP Naïve, 63% of E-Cig, and 59% of Combustibles+ had THC present in their urine.

**Figure 4:**
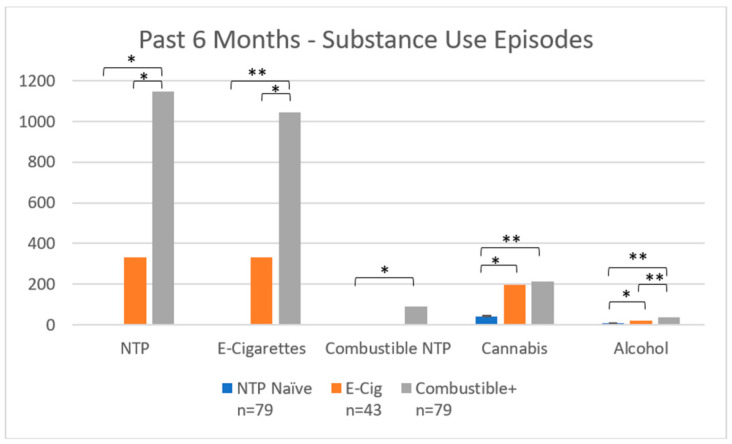
Past 6-month substance use episodes by group. Notes: * denotes *p* < 0.05; ** denotes *p* < 0.001. NTP = Nicotine and Tobacco Product. *Y*-axis displays number of NTP, cannabis, and alcohol use occasions in the past six months.

***Past 6-month substance use*:** As seen in Table 1, past six-month substance use differed by group. Combustible+ used more alcohol (F(2198) = 27.13, *p* < 0.001), e-cigarettes (F(2198) = 12.64, *p* < 0.001), cannabis (F(2198) = 7.13, *p* = 0.001), and NTP overall (F(2198) = 4.92, *p =* 0.009) than either E-Cig or NTP Naïve (see Figure 4). There were also differences in the report of high potency cannabis use, with Combustible+ and E-Cig using high potency cannabis more frequently than NTP Naïve (*p* < 0.001); yet Combustible+ and E-Cig did not differ in reported frequency of high potency cannabis use (*p* = 0.79).

**Figure 5:**
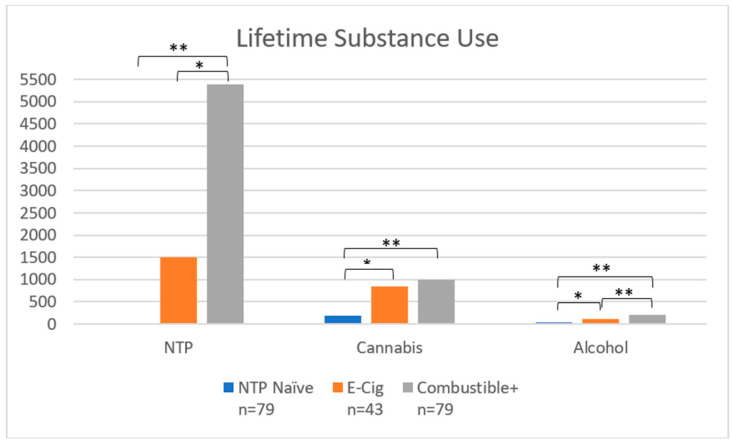
Lifetime Substance Use Episodes by Group. Notes: * denotes *p* < 0.05; ** denotes *p* < 0.001. NTP = Nicotine and Tobacco Product. *Y*-axis displays number of lifetime use occasions of NTP, cannabis, and alcohol use.

***Lifetime substance use*:** In addition, groups varied by lifetime episodes of alcohol (F(2198) = 21.48, *p* < 0.001), cannabis (F(2198) = 6.67, *p* = 0.002), and NTP use (F(2, 198) = 10.57, *p* < 0.001; see Figure 5). Combustible+ used more NTP (*p* < 0.001) and alcohol (*p* < 0.001) than E-Cig and NTP Naïve, and more cannabis than NTP Naïve (*p* < 0.001). E-Cig used more cannabis (*p* = 0.02) and alcohol (*p* = 0.045) than NTP Naïve.

***Age of onset of substance use*:** Age of onset of first use or regular use of either NTP or cannabis did not differ between NTP using groups (*p*’s > 0.05).

### 3.2. Primary Outcomes

**Figure 6:**
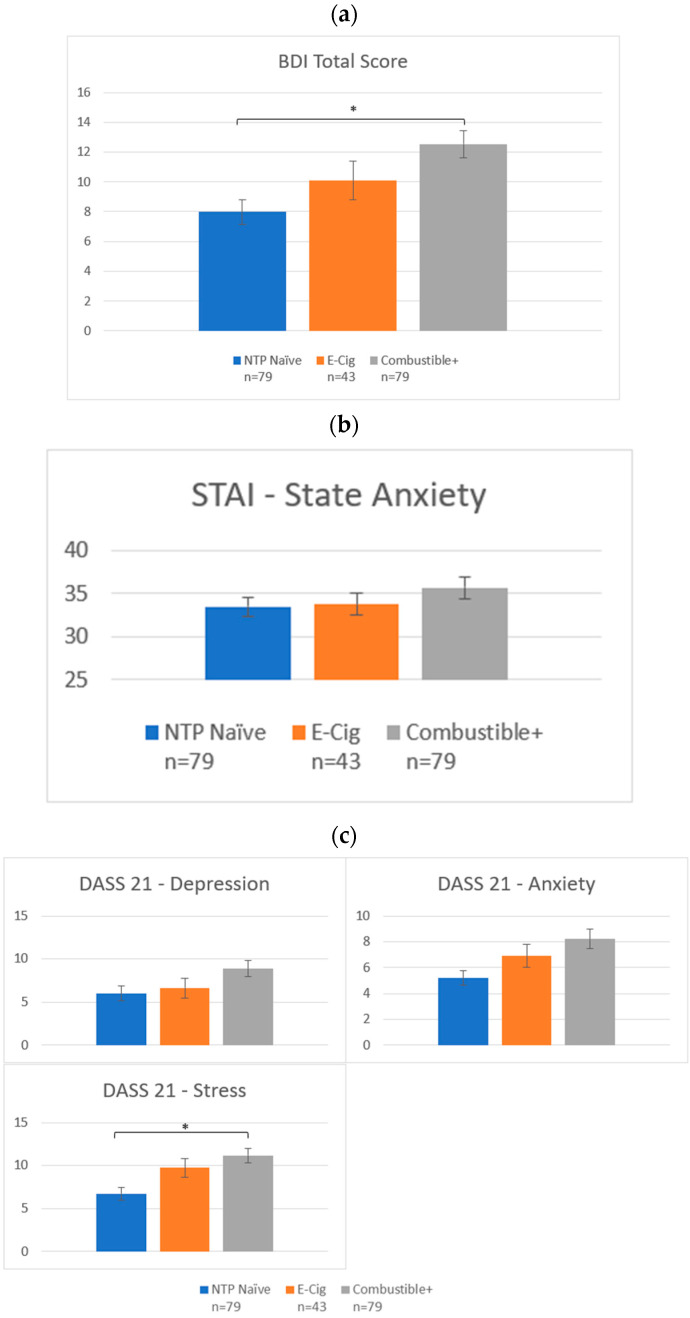
Mental health symptoms by group. (**a**) BDI-II Total Score. (**b**) STAI—State Anxiety Score. (**c**) DASS 21 Depression, Anxiety, and Stress Scores. Notes: * denotes *p* < 0.05. NTP = Nicotine and Tobacco Product. *Y*-axes display total scores on each scale and subscale.

***Mental health*:** After controlling for sociodemographic factors and past six-month alcohol and cannabis use, groups differed in total reported depressive symptoms on the BDI (F(2193) = 5.03, *p* = 0.007, FDR-*p* = 0.02). Post-hoc tests revealed that NTP Naïve reported significantly fewer depression symptoms than Combustibles+ (*p* < 0.001), with no other between group differences (see Figure 6a). The prevalence of clinical levels of depression differed between groups (χ^2^ = 6.61, *p* = 0.04), with 24% of NTP Naïve, 26% of E-Cig, and 42% of Combustible+ reporting clinical levels of depression symptoms on the BDI. In addition, females scored higher on the BDI (F(1193) = 15.16, *p* < 0.001, FDR-*p* < 0.001). Similarly, groups differed on DASS Stress Scale (F(2193) = 4.55, *p* = 0.01, FDR-*p* = 0.04; see Figure 6c), with Combustibles+ scoring higher than NTP Naïve (*p* = 0.02). Females again scored higher than males on the DASS Stress Scale (F(1193) = 11.50, *p* < 0.001, FDR-*p* < 0.001).

**Figure 7:**
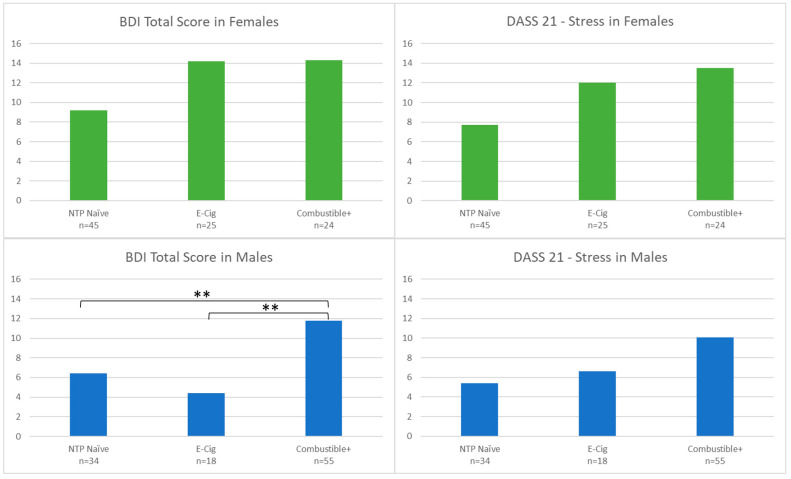
BDI Total Score and DASS 21—Stress Score by Gender and Group. Notes: ** denotes *p* < 0.001. NTP = Nicotine and Tobacco Product. *Y*-axes display total scores on each scale and subscale.

Given the significant results on BDI and DASS Stress by group status and gender, models were run separately to assess if gender moderated group differences in BDI or DASS Stress (see Figure 7). In males, BDI was significantly higher in Combustible+ as compared to the other male groups (F(2100) = 9.58, *p* < 0.001, FDR-*p* < 0.001), as was DASS Stress prior to correcting for multiple comparisons (F(2100) = 3.50, *p* = 0.03, FDR-*p* = 0.17); E-Cig and NTP Naïve did not differ from one another. In females, there was no significant difference by NTP group (BDI: *p* = 0.14; DASS Stress: *p* = 0.27).

NTP groups did not differ on any other mental health measures (STAI, DASS Depression or Anxiety subscales; see Figure 6b,c).

***Neurocognition*. *NIH Toolbox***. After controlling for past six-month alcohol and cannabis use, there were no group differences in performance across all NIH Toolbox measures: Picture Vocabulary Test (*p* = 0.63), Flanker (*p* = 0.90), List Sorting (*p* = 0.72), Card Sort (*p* = 0.30), Pattern Comparison (*p* = 0.64), Sequence Memory (*p* = 0.77), Oral Reading (*p* = 0.26). After controlling for sociodemographic factors and past six-month alcohol and cannabis use, groups did not differ on Game of Dice performance (*p* = 0.49). *RAVLT*. After controlling for sociodemographic factors and past six-month alcohol and cannabis use, groups did not differ on RAVLT initial learning (*p* = 0.79), total score (*p* = 0.88), or long delay recall (*p* = 0.65). Poorer RAVLT performance was related to being older (initial learning: F(1186) = 7.51, *p* = 0.007, FDR-*p* = 0.02; total score: F(1186) = 6.11, *p* = 0.01, FDR-*p* = 0.06; no relationship with long delay, *p* = 0.06) and using more cannabis in the past six months (initial learning: F(1186) = 6.76, *p* = 0.01, FDR-*p* = 0.02; total score: F(1186) = 6.52, *p* = 0.02, FDR-*p* = 0.06; long delay: (1186) = 4.62, *p* = 0.03, FDR-*p* = 0.19). Being more educated with associated with better initial learning (F(1186) = 7.75, *p* = 0.006, FDR-*p* = 0.02). After controlling for sociodemographic factors and past six-month alcohol and cannabis use, groups did not differ by Emotional Stroop task performance on congruent (*p* = 0.10) or incongruent (*p* = 0.17) blocks. However, better performance on congruent and incongruent blocks was associated with older age (congruent: F(1185) = 10.48, *p* = 0.001, FDR-*p* = 0.002; incongruent: F(1185) = 9.40, *p* = 0.002, FDR-*p* = 0.006) and more education (congruent: F(1185) = 12.17, *p <* 0.001, FDR-*p* < 0.001; incongruent: (F(1185) = 6.12, *p* = 0.01, FDR-*p* = 0.04), while more inaccurate performance on congruent blocks was associated with more past six-month cannabis use (F(1185) = 11.41, *p <* 0.001, FDR-*p* < 0.001).

### 3.3. Post-Hoc Analyses

Given the broad range of these participants and concerning that group differences may be due to Combustible+ users being older, analyses were re-run, excluding participants under the age of 18. These age-restricted groups did not differ by any sociodemographic factors, except for gender (χ^2^ = 12.70, *p* = 0.002). When re-running all analyses, the results remained the same with three exceptions: On the ECAS, there was no longer a group difference (*p* = 0.09). In addition to group differences by past-6-month e-cigarette, NTP, and alcohol use, Combustibles+ also used more cannabis (F(2182) = 5.62, *p* = 0.004) than controls (*p* = 0.02) but not E-Cig (*p* = 0.81). Finally, Combustible+ also used high potency cannabis more frequently (F(2124) = 5.66, *p* = 0.004) than NTP Naïve (*p* = 0.007) but not E-Cig (*p* = 0.99). All other results remained consistent with primary findings from the whole sample.

## 4. Discussion

Despite the sharp rise in emerging adult NTP use in recent years, little is known about mental health and neurocognitive differences of e-cigarette use relative to traditional combustible NTP use (e.g., tobacco cigarettes), in concert with attitude differences toward NTP use. Here, we delineate clear and significant differences in NTP users’ substance use habits and attitudes despite minimal mental health or cognitive differences by nicotine group status. There appear to be qualitative differences in motivations (self-enhancement and affect regulation), and expectancies (positive reinforcement) of smoking behavior among individuals who have recently (in the past six months) used combustible versus non-combustible products. Combustible product users at this young age also reported greater dependency on nicotine and craving for nicotine products as compared to individuals who only use e-cigarettes. Of note, individuals in both NTP groups (Combustible+ and E-Cig) had more favorable views of e-cigarettes as compared to traditional tobacco products (e-cigarette attitude survey) and largely reported using e-cigarettes for the taste. In addition, combustible product users tended to use substances more heavily overall, including more episodic NTP use. Finally, both nicotine use groups reported higher levels of depression and stress symptoms as compared to NTP naïve controls. Interestingly, when considering the moderating influence of gender, male combustible users reported more depression symptoms than the other groups, while females did not differ in depressive symptoms by group status. Though Combustible+ users were significantly older than the other groups, when restricting analyses to only those between 18 and 22 years old, results remained consistent, suggesting that it is not merely that Combustible+ users have had more time to transition into heavier substance use.

While there is a growing body of literature on susceptibility and predictors of NTP use [46], there is a paucity of knowledge about NTP attitudes in young adults who use combustible and non-combustible NTPs. Harm is often a focus, with e-cigarette users stating the belief that e-cigarettes are less harmful than combustible cigarettes [47,48,49]. However, here, we did not find a difference in perceived harm that was moderated by nicotine product use type. Motivations to use e-cigarettes in our sample tended to be more about the consideration of others (e.g., not bothering others) or alleviating dependence on combustible products, rather than about harm, cost, or other motivating factors. Combustible product users also reported combustible NTP use for its reinforcing properties, affect regulation, social facilitation, and relief of boredom.

Results suggest NTP users, and combustible product users in particular, have higher levels of substance use and more severe nicotine dependence. While alcohol and cannabis use were present in each group, E-Cig reported more use than NTP Naïve, while combustible users reported the most use. The Combustible+ group also reported more e-cigarette use than the E-Cig group and had higher levels of severity of nicotine dependence across measures. Here, it is not clear that the use of combustible NTPs are driving the attitude and/or behavioral differences, rather than other individual characteristics or pre-existing factors which impact overall substance use. This finding is in line with other reports of increased dependence in dual users of combustible and non-combustible NTP and has similarly been found in younger adolescents [22,25], in adults [23,24], in those with higher levels of tobacco product use [23], and in populations with greater substance use in general [24]. Therefore, it may be that tobacco cigarette or dual users are at increased risk of substance dependence in general and downstream negative sequelae.

NTP users in the present study reported higher levels of BDI depression and DASS stress symptoms, including a higher prevalence of symptoms crossing the threshold of clinical depression than NTP-Naïve participants. Male Combustible+ had higher levels of depression symptoms than either E-Cig or NTP Naïve, while there was no significant difference by group for females. There was also no difference in their self-reported anxiety symptoms when considering all participants together, regardless of gender. This is a cross-sectional analysis in which direction and causality cannot be determined, and therefore it is unclear if NTP use was a risk factor for depression and stress, or vice versa. Indeed, a longitudinal analysis of emerging adults found depression was an important risk factor for nicotine dependence [50], while another longitudinal study found NTP use was associated with later depression [51]. Others have found cigarette, but not e-cigarette use, linked to mental health functioning [52]. Given preliminary evidence here of heightened risk for combustible use among males with elevated depression scores, gender and emotional functioning should continue to be monitored and examined as potential risk factors for combustible product use. Future research is necessary to disentangle modes of nicotine administration and mental health outcomes in adolescents and emerging adults.

This investigation is the first known study of e-cigarette use that objectively measured neurocognitive performance, and no differences in cognition by nicotine group status were observed. Cognitive differences in adolescent and young adult NTP users have been noted previously [13,14,53], though not always [53,54]. E-cigarettes specifically have been linked to poorer self-reported neurocognition [20,21], though subjective concerns may not relate to true performance deficits. Lower or more acute doses of nicotine are linked to cognitive enhancement, while chronic and/or high doses of nicotine are linked to desensitization of nicotine acetylcholine receptors (nAChRs), including alterations of the modulation of dopamine, serotonin, and other receptors, which potentially impact cognitive performance [55]. While 12 h of abstinence from alcohol, cannabis, and all other drug use were required for the present study, participants were allowed to smoke/vape during their study session to prevent withdrawal effects and, therefore, potentially enhance neurocognitive performance. Our participants were also using NTPs at relatively low levels in the past six months, vaping three-to-four times a day on average and, in combustible users, smoking combustible products five-to-six days per month. The lack of differences may imply that relatively low level NTP use may not be as detrimental as previously thought, suggesting that there is still time for intervention before more of the negative sequelae of sustained nicotine use and dependence become apparent.

Interestingly, while not a primary aim of the present study, past six-month cannabis use was related to poorer Emotional Stroop congruent emotion processing (accurately identifying word–facial expression pairings), with no difference on the noncongruent condition. Congruent processing accuracy is particularly relevant for processing speed and attention, deficits previously shown in cannabis users [56,57,58] and in pre-adolescent youth with higher externalizing symptoms [41]. Further, prior research indicates cannabis users may be particularly vulnerable to cognitive control and affective processing deficits, including when identifying emotional faces [59]. Cannabis users may have to use more neural resources to achieve the same level of performance [60], though this may not happen as readily on a less demanding task, such as on a congruent processing task. Future research should continue to investigate socioaffective response in cannabis users.

Limitations: All groups included alcohol and cannabis use which, while better generalizing to typical real-world use patterns, may limit ability to detect differences due to NTP use alone, despite attempts to statistically control for these substances in the analyses. In addition, both E-Cig and Combustible+ used e-cigarette products, which may also be a source of bias, and makes it difficult to disentangle findings that are unique to e-cig vs. combustible product use. As mentioned above, NTP users in this age range, and particularly combustible NTP users, may not yet be using at a level to meaningfully impact neurocognition or mental health. Alternatively, the lower level of use may have contributed to limited neurocognitive differences in this young adult sample. Though group differences in attitudes are noted, and data are descriptive, it is unclear what individual and environmental characteristics may contribute to the acquisition of these attitudes. Finally, the present analyses are cross sectional in nature; longitudinal studies designed for causal inference are needed to establish directionality of results.

The present findings add to the field’s understanding of the unique and shared characteristics between adolescent and young adult combustible and non-combustible NTP users. Combustible cigarette users have different motivations for e-cigarette use, and greater severity of nicotine dependence. They also use more NTP, cannabis, and alcohol use than non-combustible e-cigarette users or NTP naïve participants. While there were no differences in cognitive performance by nicotine group status, both nicotine groups reported higher levels of depression than those who were NTP naïve. In addition, male combustible users had higher levels of depression than male non-combustible users or naïve individuals. Taken together, individuals ages 16–22 are still using combustible products and those who use combustible NTP at any levels are likely qualitatively different than non-combustible using peers and may be more vulnerable to poorer health outcomes. Future research in our laboratory will investigate differences in trajectories of combustible NTP users and consider other potential brain-behavior outcomes as related to combustible and non-combustible NTP use.

## Figures and Tables

**Table 1:** Demographics and substance use characteristics.

	NTP Naïve (*n* = 79) M/% (SD) *Range*	E-Cig (*n* = 43) M/% (SD) *Range*	Combustibles+ (*n* = 79) M/% (SD) *Range*	*p-*Value
Age (Yrs)	18.96 (1.66) *16–22*	19.42 (1.68) *17–22*	19.94 (1.30) *18–22*	**<0.001**
Education (Yrs)	12.68 (1.64) *9–16*	13.07 (1.42) *10–16*	13.24 (1.20) *10–15*	**0.048**
Mother’s Education (% Bachelor’s or above)	47%	53%	49%	0.80
% Female	57%	58%	30%	**<0.001**
% Hispanic	43%	33%	38%	0.52
% Caucasian	39%	49%	62%	0.06
Past 6-month e-cig use (total use episodes)	-	330.93 (716.96) *1–3585*	1046.62 (2047.57) *1–10,244*	**<0.001**
Past 6-month cigarette use (total use episodes)	-	-	79.16 (421.46) *0–3620*	0.12
Past 6 month any combustible use (total use episodes)	-	-	90.04 (427.96) 1–362	0.07
Age of first NTP use	16.85 (1.86) 13–20 *n* = 13	16.65 (1.88) *9–20*	16.66 (1.93) *12–21*	0.94
Age of first regular NTP use	15 *n* = 1	18.60 (1.68) *15–22* *n* = 25	17.86 (1.82) *14–21* *n* = 56	0.03
% No Alcohol	32%	0%	0%	**<0.001**
Past 6-month alcohol use (total use episodes)	8.84 (14.64) *0–75* *n* = 54	19.79 (19.72) *0.5–75* *n* = 43	35.62 (30.66) *2–145* *n* = 79	**<0.001**
% No Cannabis	49%	12%	4%	**<0.001**
Past 6-month cannabis use (total use episodes)	43.15 (91.36) *0–474* *n* = 40	198.68 (254.47) *0–1035* *n* = 38	214.68 (439.04) *0–3557* *n* = 76	**0.001**

Notes: sample size (*n*) indicated for number of participants who endorsed a response when it was not endorsed by all participants with one or both groups (e.g., not all participants had used NTP regularly). Bolded *p*-values indicate *p* < 0.05.

**Table 2:** Reasons for e-cigarette use.

	E-Cig (*n* = 43) %	Combustibles+ (*n* = 79) %	χ^2^	*p*-Value
**I believe it is less harmful to my health**	44%	44%	<0.001	*0.99*
**I believe it is less harmful to others**	42%	39%	0.08	*0.78*
**I prefer the taste of an e-cigarette**	58%	56%	0.07	0.80
**E-cigarettes are less toxic than tobacco**	44%	37%	0.65	*0.42*
**To deal with craving for tobacco**	5%	16%	3.60	*0.06*
**To quit smoking or avoid relapsing**	0%	9%	4.04	*0.04*
**To deal with withdrawal symptoms**	2%	13%	3.62	*0.06*
**E-cigarettes are cheaper than smoking**	14%	23%	**1.37**	*0.24*
**To avoid bothering others with tobacco**	9%	34%	9.09	*0.003*
**To deal with situations where I can’t smoke (e.g., at work)**	23%	32%	0.96	*0.33*
**To avoid having to go outside to smoke**	9%	47%	17.58	*<0.001*
**To reduce tobacco consumption in preparation of a quit attempt**	5%	11%	1.54	*0.21*
**To reduce tobacco consumption with NO intention to quit smoking**	0%	15%	7.24	*0.007*
**Unable to stop using it**	7%	19%	3.73	*0.05*

## Data Availability

Data available on request due. The data presented in this study are available on request from the corresponding author.
